# Mycobacterium tuberculosis Rv0991c Is a Redox-Regulated Molecular Chaperone

**DOI:** 10.1128/mBio.01545-20

**Published:** 2020-08-25

**Authors:** Samuel H. Becker, Kathrin Ulrich, Avantika Dhabaria, Beatrix Ueberheide, William Beavers, Eric P. Skaar, Lakshminarayan M. Iyer, L. Aravind, Ursula Jakob, K. Heran Darwin

**Affiliations:** a Department of Microbiology, New York University School of Medicine, New York, New York, USA; b Department of Molecular, Cellular, and Developmental Biology, University of Michigan, Ann Arbor, Michigan, USA; c Proteomics Laboratory, Division of Advanced Research Technologies, New York University School of Medicine, New York, New York, USA; d Vanderbilt Institute of Infection, Immunology, and Inflammation, Vanderbilt University Medical Center, Nashville, Tennessee, USA; e National Center for Biotechnology Information, National Library of Medicine, National Institutes of Health, Bethesda, Maryland, USA; National Cancer Institute

**Keywords:** Hsp70, *Mycobacterium*, chaperone, protein, proteostasis, tuberculosis

## Abstract

The bacterial pathogen Mycobacterium tuberculosis is the leading cause of death by an infectious disease among humans. Here, we describe a previously uncharacterized M. tuberculosis protein, Rv0991c, as a molecular chaperone that is activated by oxidation. Rv0991c has homologs in most bacterial lineages and appears to function analogously to the well-characterized Escherichia coli redox-regulated chaperone Hsp33, despite a dissimilar protein sequence. Rv0991c is transcriptionally coregulated with *hsp60* and *hsp70* chaperone genes in M. tuberculosis, suggesting that Rv0991c functions with these chaperones in maintaining protein quality control. Supporting this hypothesis, we found that, like oxidized Hsp33, oxidized Rv0991c prevents the aggregation of a model unfolded protein *in vitro* and promotes its refolding by the M. tuberculosis Hsp70 chaperone system. Furthermore, Rv0991c interacts with DnaK and can associate with many other M. tuberculosis proteins. We therefore propose that Rv0991c, which we named “Ruc” (redox-regulated protein with unstructured C terminus), represents a founding member of a new chaperone family that protects M. tuberculosis and other species from proteotoxicity during oxidative stress.

## Abstract (precis)

M. tuberculosis infections are responsible for more than 1 million deaths per year. Developing effective strategies to combat this disease requires a greater understanding of M. tuberculosis biology. As in all cells, protein quality control is essential for the viability of M. tuberculosis, which likely faces proteotoxic stress within a host. Here, we identify an M. tuberculosis protein, Ruc, that gains chaperone activity upon oxidation. Ruc represents a previously unrecognized family of redox-regulated chaperones found throughout the bacterial superkingdom. Additionally, we found that oxidized Ruc promotes the protein-folding activity of the essential M. tuberculosis Hsp70 chaperone system. This work contributes to a growing body of evidence that oxidative stress provides a particular strain on cellular protein stability.

## INTRODUCTION

The folding of a protein that is in a nonnative conformation, including during translation or upon stress-induced denaturation, can be accomplished in all organisms by a set of chaperones belonging to the Hsp70 and Hsp40 protein families. In bacteria, these chaperones are called DnaK and DnaJ, respectively (reviewed in reference 1). DnaK is an ATPase that iteratively binds to and releases protein substrates, allowing them to fold into a native conformation. In the ATP-bound state, DnaK has a low substrate affinity. Engagement of DnaK with a substrate induces ATP hydrolysis in a DnaJ-dependent manner, resulting in an ADP-bound DnaK-substrate complex. Finally, replacement of ADP with ATP results in release of the client protein, which has either reached its native structure or can rebind to DnaK (2, 3). This cycle of high- and low-affinity substrate-binding states is facilitated by a nucleotide exchange factor, known in bacteria as GrpE (4). Besides harboring intrinsic protein-folding activity, the DnaK/DnaJ/GrpE system (DnaKJE) can also deliver substrates to GroEL/GroES chaperonins (also known as Hsp60/Hsp10) (5, 6), and prokaryotic and eukaryotic DnaKJE homologs can cooperate with proteases to promote the degradation of unfolded substrates (7, 8).

Proteins in nonnative conformations often expose hydrophobic regions that are prone to aggregation, an event that is toxic to cells. Importantly, aggregate formation becomes irreversible if DnaKJE cannot access unfolded proteins for refolding. To remedy this situation, the Hsp100 family of ATP-dependent chaperones (called ClpB in bacteria) cooperate with DnaKJE by solubilizing proteins within aggregates (9–11). The prevention of irreversible protein aggregation is also accomplished in all organisms by the small heat shock protein (sHsp) family of chaperones. Rather than actively dissociating protein aggregates by ATP hydrolysis, sHsps act by simply binding to denatured or misfolded proteins; the presence of sHsps within protein aggregates allows for efficient refolding by ATPase chaperones (5, 12–15). Thus, in contrast to “refoldases,” sHsps are “holdases” that afford cells the ability to rapidly respond to proteotoxic stress in an energy-independent manner.

While DnaKJE, chaperonins, ClpB, and sHsps are all active under steady-state conditions, there are also noncanonical chaperones that only become active upon encountering specific stresses. Hsp33 (encoded by *hslO*), found in both prokaryotes and eukaryotes, and the eukaryotic Get3 are inactive as chaperones in the normal reducing environment of the cytoplasm; however, oxidation of cysteine thiols within Hsp33 or Get3 induces conformational changes that allow them to bind to unfolded proteins (6, 16–19). Hsp33 prevents the irreversible aggregation of unfolded proteins by delivering substrates to ATPase chaperones for refolding after stress is alleviated (6). In Escherichia coli, this activity is of particular importance during oxidative stress, during which low ATP levels can cause DnaK to enter a partially unfolded, nucleotide-depleted state; the presence of Hsp33 allows for refolding by DnaKJE to proceed once a normal temperature and reducing environment are restored (20). Two other bacterial chaperones, RidA and CnoX, are activated by the oxidant hypochlorous acid through a mechanism that does not involve oxidation of cysteine thiols but, rather, chlorination of free amino groups (21, 22). Aside from oxidation, other noncanonical chaperones have been found to activate upon exposure to high temperatures or acid (reviewed in reference 23).

The bacterial pathogen Mycobacterium tuberculosis is currently responsible for the majority of human infectious disease-related deaths worldwide (24). In this work, we describe our discovery that the uncharacterized M. tuberculosis gene Rv0991c encodes a chaperone, which we named Ruc (redox-regulated chaperone with unstructured C terminus). Ruc belongs to a previously unacknowledged but evolutionarily widespread family of bacterial proteins with little predicted structural similarity to other chaperones. Upon oxidation, Ruc is capable of inhibiting protein aggregation and can promote the refolding of unfolded proteins by DnaKJE. Ruc was also found to interact with DnaK in M. tuberculosis; however, despite its association with this essential protein-folding chaperone, Ruc did not have a strong role for M. tuberculosis virulence in mice and was not required for bacterial survival during *in vitro* oxidative stress under several conditions tested. Taken together, these observations suggest that Ruc is important for the ability of M. tuberculosis and many other bacterial species to withstand oxidation-associated proteotoxicity under to-be-determined conditions.

## RESULTS

### Ruc induction during heat stress requires the M. tuberculosis Pup-proteasome system.

**FIG 1:**
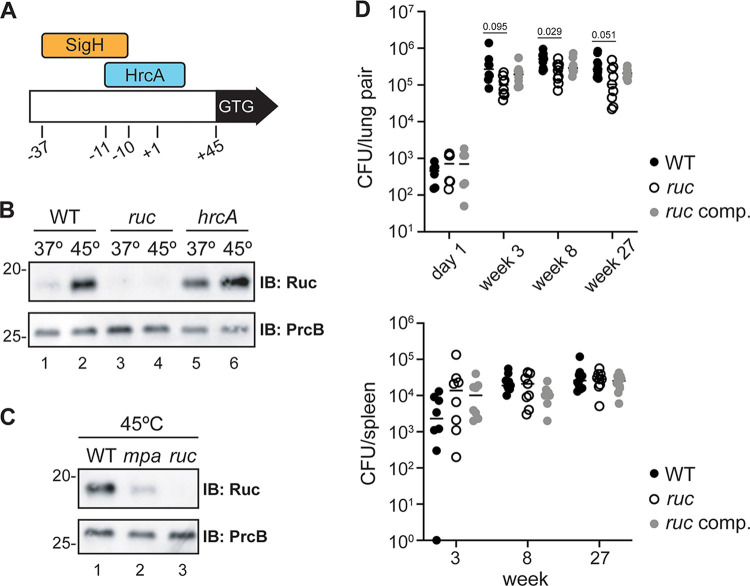
M. tuberculosis Ruc is a heat shock-inducible, HrcA- and Mpa-regulated small protein. (A) Illustration of the *ruc* control region in M. tuberculosis. The position of the *ruc* transcriptional start site (+1), as well as the binding sites of sigma factor SigH and repressor HrcA, is shown relative to the +1 (26, 27). A second +1 was identified 19 nucleotides upstream of the +1 shown here (71). (B) WT (MHD1), *ruc* (MHD1384), and *hrcA* (MHD1384) M. tuberculosis strains (see Table 1 for details) were incubated at 37°C or 45°C, and Ruc abundance was assessed in bacterial lysates by immunoblotting (IB). Immunoblotting for PrcB was used as a loading control. (C) WT, *mpa* (MHD149), and *ruc* strains analyzed as in panel B. (D) Mice were infected with WT with empty vector (MHD1385), *ruc* with empty vector (MHD1393), or *ruc* complemented with pMV306kan-*ruc* (MHD1394) M. tuberculosis strains, and the bacterial burden in the lungs (top) and spleen (bottom) was determined by the number of CFU at day 1 or at weeks 3, 8, and 27 postinfection. Statistical significance for the CFU differences between strains at each time point were calculated using one-way analysis of variance (ANOVA); the *P* values approaching statistical significance are shown above the data points. All other data points had *P* values of >0.1. Data represent the combined results of two independent infection experiments.

We became interested in Ruc after identifying the transcriptional repressor HrcA as a putative target of the M. tuberculosis Pup-proteasome system (25). In M. tuberculosis, HrcA directly represses four genes; three of them encode highly conserved chaperone proteins of the Hsp60/Hsp10 family, while the fourth gene, *ruc*, encodes a protein of unknown function (26). In addition to its negative regulation by HrcA, *ruc* is induced during heat stress by SigH, a sigma factor that also activates transcription of the Hsp70/Hsp40 genes (27). The DNA sequences to which SigH and HrcA bind upstream of the *ruc* start codon overlap, suggesting that these two transcriptional regulators compete for binding to the *ruc* promoter (Fig. 1A). We compared Ruc abundance upon incubation of M. tuberculosis at 37°C or 45°C and found that Ruc levels increased dramatically at 45°C, consistent with previously reported transcriptional data (Fig. 1B, lanes 1 and 2; see Table 1 for strains) (26). The low abundance of Ruc at 37°C is primarily due to repression by HrcA, given that Ruc was highly abundant in an *hrcA* mutant at this temperature (Fig. 1B, lane 5). We observed a further induction of Ruc levels during heat shock in an *hrcA* mutant (Fig. 1B, lane 6). These results corroborate earlier evidence that *ruc* expression is controlled in two ways: through repression by HrcA and induction by SigH.

**TABLE 1:** Strains, plasmids, and primers used in this work

Strain, plasmid, or primer	Relevant genotype, description, or sequence	Source or reference no.
*M. tuberculosis* strain		
MHD1	Wild-type H37Rv	ATCC 25618
MHD1383	Hyg^r^; Δ*hrcA*::*hyg*	25
MHD1384	Hyg^r^; Δ*ruc*::*hyg* (deletion-disruption mutation of Rv0991c)	25
MHD149	Hyg^r^; Δ*mpa*::*hyg*	61
MHD1541	Hyg^r^; H37Rv, pOLYG-*ruc*-TAP	This study
MHD1385	Kan^r^; H37Rv, pMV306kan	This study
MHD1393	Kan^r^, Hyg^r^; MHD1384, pMV306kan	This study
MHD1394	Kan^r^, Hyg^r^; MHD1384, pMV306kan-*ruc*	This study
*E. coli* strain		
DH5α	*supE44* Δλ*acU169* (φ*80 lacZ*Δ*M15*) *hsdR17 recA1 endA1 gyrA96 thi-1 relA1* (Nal^r^)	Gibco
ER2566	F-λ-f*huA2* (*lon*) *ompT lacZ::T7 geneI gal sulA11* Δ(m*crC-mrr*)1*14::IS10 R*(*mcr-73*::miniTn*10*)2 *R*(*zgb-210*::Tn*10*)1 (tetS) *endA1* (*dcm*)	72
BL21(DE3)	F^–^ *ompT gal dcm lon hsdS_B_*(*r_B_*^−^*m_B_*^–^) λ(DE3 [*lacI lacUV5*-*T7p07 ind1 sam7 nin5*] (*malB*^+^)_K-12_(λ^S^)	New England Biolabs
Plasmid		
pET24b(+)	Kan^r^; for inducible production of recombinant protein in *E. coli*	Novagen
pET24b(+)-Rv0991c-his6	Kanr; for production of recombinant Ruc with C-terminal His_6_ (Ruc-His_6_)	This study
pAJD107	Amp^r^; contains multiple restriction sites for cloning	73
pAJD107-Rv0991c	Amp^r^; for making the *ruc* expression vector	This study
pAJD107-Rv0991c-C8SC11S	Amp^r^; for cloning HisSUMO-*ruc-*C8SC11S expression vector	This study
pET24b(+)-Rv0991c	Kan^r^; for production of recombinant, native Ruc	This study
pET24b(+)-Ruc-C8,11S	Kan^r^; for cloning `HisSUMO-*ruc-*C8SC11S expression vector	This study
pEcTL02	Amp^r^; for purification of *M. tuberculosis* ClpB with N-terminal His_6_-SUMO from *E. coli*	12
pEcTL04	Amp^r^; for purification of *M. tuberculosis* DnaJ2 with N-terminal His_6_-SUMO from *E. coli*	12
pEcTL05	Amp^r^; for purification of *M. tuberculosis* GrpE with N-terminal His_6_-SUMO from *E. coli*	12
pEcTL06	Amp^r^; for purification of *M. tuberculosis* DnaK with N-terminal His_6_-SUMO from *E. coli*	12
pET24b(+)-HisSUMO-*ruc*	Kan^r^; for purification of *M. tuberculosis* Ruc with N-terminal His_6_-SUMO from *E. coli*	This study
pET24b(+)-HisSUMO-*ruc-*Nterm	Kan^r^; for purification of *M. tuberculosis* Ruc_Nterm_ (amino acids 1 through 49) with N-terminal His_6_-SUMO from *E. coli*	This study
pET24b(+)-HisSUMO-*ruc-*C8SC11S	Kan^r^; for purification of *M. tuberculosis* Ruc_C8S,C11S_ (containing cysteine to serine substitutions in residues 8 and 11) with N-terminal His_6_-SUMO from *E. coli*	This study
pET24b(+)-HisSUMO-*ruc-*C29S,C32S	Kan^r^; for purification of *M. tuberculosis* Ruc_C29S,C32S_ (containing cysteine to serine substitutions in residues 29 and 32) with N-terminal His_6_-SUMO from *E. coli*	This study
pHYRS52	Amp^r^; for purification of SUMO protease (*S. cerevisiae* Ulp1, amino acids 403 to 621) with N-terminal His_6_ from *E. coli*	Addgene
pOLYG	Hyg^r^; for overproduction of proteins in *M. tuberculosis*	74
pOLYG-Rv0991c-TAP	Hyg^r^; for purification of Ruc with C-terminal hexahistidine-FLAG tandem affinity purification tag from *M. tuberculosis*	This study
pMV306kan	Kan^r^; for integration into the L5 *attB* site of the *M. tuberculosis* chromosome	75
pMV306kan-Rv0991c	Kan^r^; *ruc* complementation plasmid	This study
Primer		
NdeI-Rv0991c-F	gatcCATATGccaacctacagctacgagtgcacc	
HindIII-Rv0991c-R	gtagAAGCTTgacggccgcggcggcg	
HindIII-Rv0991c-F	gtagAAGCTTtcgtctagtcgcggtggtgcg	
XbaI-Rv0991c-R	ttatTCTAGAtcagacggccgcggcgg	
XbaI-Rv0991c-TAP-R	gatcTCTAGAtcagtggtggtggtggtggtgctcgagtgcggccgccttatcgtcgtcatccttgtaatcgacggccgcggcggcggttgtgga	
BglII-Rv0991c-R	tagacAGATCTtcagacggccgcggcggcggttgt	
SUMO-Ruc-soeR	ctcgtagctgtaggttggcaccccaccaatctgttctctgtgagcctc	
SUMO-Ruc-soeF	gaggctcacagagaacagattggtggggtgccaacctacagctacgag	
Ruc-KpnI-R	tataGGTACCtcagacggccgcggcggcggttgt	
RucNterm-KpnI-R	tataGGTACCtcagcctttgaacaccacgccgaccgc	
NdeI-Rv0991c-C8SC11S-F	GCGCCATATGCCAACCTACAGCTACGAGAGCACCCAGAGCGCCAACCGCTTCGATGTTGTG	
Rv0991c-C29SC32S-F	CCGACGATGCGCTGACCACGAGCGAGCGGAGTTCTGGCCGGCTGCGCAAGCTGTTC	
Rv0991c-C29SC32S-R	GAACAGCTTGCGCAGCCGGCCAGAACTCCGCTCGCTCGTGGTCAGCGCATCGTCGG	
T7-F	taatacgactcactataggg	
T7-term	GCTAGTTATTGCTCAGCGG	

In a recent study, we proposed that the pupylation and degradation of HrcA by the M. tuberculosis proteasome is required for the full expression of the HrcA regulon. We previously reported the abundance of HrcA-regulated gene products between a wild-type (WT) M. tuberculosis strain and an *mpa* mutant, which cannot degrade pupylated proteins, and found that GroES, GroEL1, and GroEL2 levels are significantly lower in an *mpa* strain; however, Ruc abundance is unaffected by disruption of *mpa* under these conditions. This result suggested that proteasomal degradation of HrcA is not sufficient for Ruc production under the conditions tested (25); however, this experiment was performed with cultures incubated at 37°C in minimal medium. We therefore compared Ruc abundance from the same strains incubated at 45°C and observed a striking defect in Ruc production in the *mpa* mutant (Fig. 1C, lane 2). This result supports the hypothesis that the proteasomal degradation of HrcA is required for M. tuberculosis to robustly induce *ruc* expression during heat stress.

### A *ruc* null mutant does not have a strong virulence defect in C57BL/6J mice.

Previous studies have shown that M. tuberculosis protein quality control pathways are important for its pathogenesis. M. tuberculosis strains deficient in *clpB* or the sHsp-encoding *acr2* have impaired virulence in mice (28, 29), while a mutant lacking HspR, which represses the expression of *clpB*, *acr2*, and the *hsp70/hsp40* genes, also produces less severe infections (30). Because *ruc* is coregulated with essential chaperones, we tested if Ruc was required for the full virulence of M. tuberculosis by inoculating C57BL/6J mice with our M. tuberculosis strains by aerosol and assessing bacterial burden in the lungs and spleens at several time points following infection. We performed the entire experiment twice and combined the data from both sets of infections. In the lungs, a *ruc* mutant had a subtle growth defect compared to the WT and complemented strains, however, this phenotype was not statistically significant at most time points (*P > *0.05) (Fig. 1D, top). Furthermore, we observed no differences in survival among the strains in the spleens (Fig. 1D, bottom). Based on these data, we concluded that Ruc does not play an essential role for M. tuberculosis virulence in this mouse infection model.

### Ruc is part of a novel protein family found across the bacterial superkingdom.

According to the mycobacterial genome database MycoBrowser, Ruc is conserved in both pathogenic and nonpathogenic mycobacteria (31). To determine the broader phyletic distribution of Ruc among bacteria, we searched the Ruc protein sequence against a curated collection of 7,423 complete prokaryotic genomes. Ruc is highly conserved throughout the *Planctomycetes*, *Chloroflexi*, Deltaproteobacteria and *Betaproteobacteria*, *Actinobacteria*, *Spirochaetes*, and *Verrucomicrobia* lineages; additionally, Ruc is found in species within other major bacterial lineages such as *Gammaproteobacteria*, *Firmicutes*, and *Cyanobacteria*. This phyletic pattern suggests that Ruc was present in the last bacterial common ancestor, with rare lateral transfers to archaea (Table 2).

**TABLE 2:** Occurrence of *ruc* in bacterial and archaeal phyla

Phylum (no. of genomes)	% genomes with *ruc*
Bacteria	
*Gammaproteobacteria* (923)	26.65
*Betaproteobacteria* (333)	82.88
*Zetaproteobacteria* (5)	80
*Alphaproteobacteria* (469)	17.27
*Deltaproteobacteria* (86)	86.05
*Proteobacteria* (43)	74.42
*Thermodesulfobacteria* (4)	100
*Spirochaetes* (70)	48.57
*Deferribacteres* (4)	100
*Chrysiogenetes* (1)	100
*Nitrospirae* (73)	86.3
*Acidobacteria* (9)	100
*Elusimicrobia* (3)	33.33
*Verrucomicrobia* (74)	60.81
*Chlamydiae* (43)	6.98
*Planctomycetes* (18)	100
*Bacteroidetes* (263)	1.9
*Chlorobi* (263)	5.7
*Fibrobacteres* (2)	0
*Gemmatimonadetes* (3)	100
*Ignavibacteriae* (2)	100
*Aquificae* (15)	20
*Dictyoglomi* (2)	100
*Thermotogae* (29)	100
*Deinococcus-Thermus* (26)	100
*Synergistetes* (5)	0
*Fusobacteria* (16)	0
*Thermobaculum* (1)	100
*Actinobacteria* (497)	73.84
*Chloroflexi* (22)	100
*Armatimonadetes* (15)	73.33
*Tenericutes* (124)	0
*Firmicutes* (772)	8.94
*Cyanobacteria* (127)	13.39
*Calditrichaeota* (2)	100
Unclassified (2,565)	11.89
Archaea	
*Crenarchaeota* (61)	1.64
*Euryarchaeota* (187)	6.42
*Archaea* (31)	3.23

**FIG 2:**
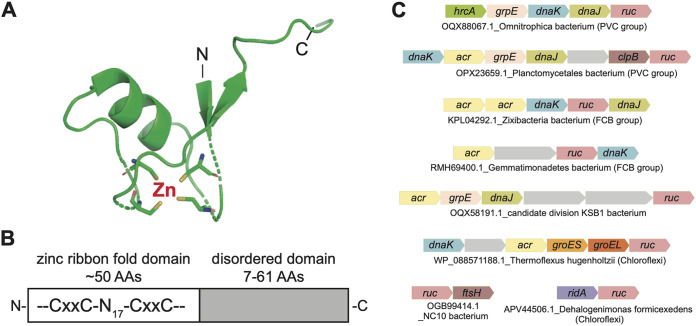
Ruc contains a putative zinc-binding domain with a disordered C terminus and cooccurs with proteostasis genes in diverse bacterial lineages. (A) Predicted structure of the N-terminal region of M. tuberculosis Ruc (residues 5 to 48) based on Phyre2 predictive modeling (32). The four cysteines in Ruc are represented as sticks, with the thiol groups shown in yellow. The position of a predicted zinc ion is also shown. (B) Illustration of the conserved features of Ruc. x, unspecified amino acid; N_17_, region 17 residues in length. (C) Genetic loci containing *ruc* with neighboring genes encoding chaperones, proteases, or chaperone-associated transcriptional regulators. Representatives of the phyletic groups PVC and FCB, as well as members of *Chloroflexi* and unclassified phyla, are shown. Genes are represented by the NCBI GenBank database accession number of the *ruc* gene followed by the species name and bacterial clade in bracket (if known).

To define the conserved features of Ruc, we used Phyre2, a program that predicts secondary and tertiary protein structures based on published sequences and solved structures (32). Alignment of Ruc with its homologs in other bacteria identified two conserved domains. The amino (N)-terminal region, consisting of approximately 50 amino acids, contains four cysteine (Cys) residues arranged in a manner consistent with a zinc ribbon fold, a domain found in zinc-binding proteins of highly diverse functions (Fig. 2A) (33). The carboxyl (C)-terminal regions of Ruc homologs consist of highly variable sequences of amino acids ranging in length from approximately 7 to 61 residues. While these C-terminal domains do not share significant similarity at the protein sequence level, they appear to be uniformly composed of hydrophilic residues with no predicted secondary structure. Overall, these structural predictions allowed us to conclude that M. tuberculosis Ruc and its homologs are likely characterized by a metal-coordinating N-terminal domain and a disordered C terminus (Fig. 2B).

Having established that *ruc* is coexpressed with the Hsp60 and Hsp70 chaperone system genes in M. tuberculosis and is present in diverse bacterial lineages, we next asked if *ruc* is associated with protein quality control genes in other species. A comprehensive gene neighborhood analysis among bacteria recovered widespread associations with chaperone genes, as well as transcription factors and proteases that regulate chaperone production. In 32% of the PVC (*Planctomycetes*, *Verrucomicrobia*, and *Chlamydiae*) and in 21% of the FCB (*Fibrobacteres*, *Chlorobi*, and *Bacteroidetes*) bacterial superphyla, *ruc* is present in loci encoding DnaK, DnaJ, GrpE, ClpB, alpha-crystallin sHsps (Acr), and HrcA. In bacteria from the *Chloroflexi* and *Gemmatimonadetes* phyla, as well as in several unclassified species, *ruc* is additionally associated with genes encoding GroES/GroEL chaperonins, the chaperone-regulating membrane protease FtsH, and the redox-regulated chaperone RidA (Fig. 2C). The identification of these conserved genetic linkages across a wide variety of phyla, as well as the observation that *ruc* is transcriptionally coregulated with essential chaperone genes in M. tuberculosis, strongly suggested that Ruc performs a function related to protein folding.

### Ruc coordinates zinc and is an intrinsically disordered protein.

Based on the observations that *ruc* is closely associated with essential chaperone genes and that Ruc contains putative zinc-coordinating cysteines, we hypothesized that Ruc has oxidation-dependent chaperone activity. Hsp33 and Get3, two well-characterized redox regulated chaperones, each contain a zinc-coordinating domain consisting of four cysteines. Under oxidizing conditions, these cysteines form intramolecular disulfide bonds and release zinc, allowing these chaperones to form complexes with denatured proteins to prevent their irreversible aggregation (16, 18, 34).

**FIG 3:**
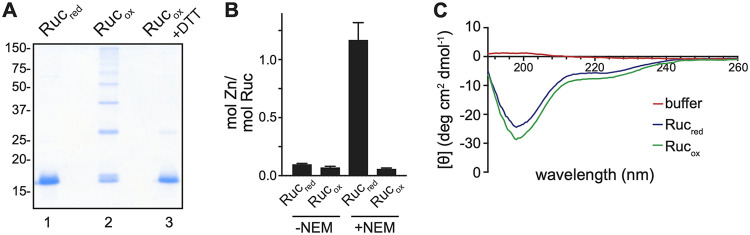
Ruc contains redox-active cysteines that coordinate a single zinc atom and is an intrinsically disordered protein. (A) Ruc_red_, Ruc_ox_, or Ruc_ox_ treated with the thiol-reducing agent DTT were separated on an SDS-PAGE gel and stained with Coomassie brilliant blue. (B) Zinc coordination by Ruc_red_ or Ruc_ox_ was quantified in the absence or presence of NEM, which modifies cysteines. Zinc concentrations were measured spectrophotometrically using the metal chelator PAR (see Materials and Methods for details). (C) Assessment of Ruc secondary structure using circular dichroism, with degrees of ellipticity (θ) plotted by wavelength.

To begin to test Ruc chaperone activity using *in vitro* assays, we produced and purified M. tuberculosis Ruc from E. coli under reducing conditions (Ruc_red_). We oxidized purified Ruc (Ruc_ox_) by incubation with hydrogen peroxide (H_2_O_2_) and copper chloride (CuCl_2_), which react to generate hydroxyl radicals that rapidly oxidize cysteines (35). On an SDS-PAGE gel, Ruc_red_ migrated as a single band (Fig. 3A, lane 1), while Ruc_ox_ migrated through the gel as multiple species whose sizes were consistent with the formation of covalent, intermolecular disulfide bonds (Fig. 3A, lane 2). Treatment of Ruc_ox_ with the thiol-reducing agent dithiothreitol (DTT) resulted in a significant loss of higher-molecular-weight species, indicating that the oxidation of Ruc cysteines is reversible (Fig. 3A, lane 3). These results demonstrate that Ruc cysteines form disulfide bonds under oxidizing conditions to create covalently linked multimers.

In Hsp33, four conserved cysteines coordinate zinc. This zinc binding maintains Hsp33 in an inactive state yet allows Hsp33 to become readily activated once an oxidant is present (34). To determine if Ruc binds zinc using its four cysteines, we used 4-(2-pyridylazo)resorcinol (PAR), a chemical that chelates free zinc to yield an absorbance peak at 500 nm (A_500_) (see Materials and Methods). Incubation of either Ruc_red_ or Ruc_ox_ with PAR alone did not yield a significant change in A_500_, demonstrating that the protein preparations contained little free zinc; however, addition of *N*-ethylmaleimide (NEM), which forms adducts on cysteine thiols, resulted in the release of zinc from Ruc_red_ that could be detected in approximately equimolar abundance to Ruc_red_. Meanwhile, addition of NEM to Ruc_ox_ did not reveal any change in zinc levels (Fig. 3B). These data suggest that under reducing conditions, Ruc cysteines coordinate an atom of zinc and that this binding is disrupted when the cysteine thiols are oxidized.

Given that PAR can chelate metals in addition to zinc (36), we next determined the precise identity of the metal bound to Ruc using inductively coupled plasma mass spectrometry (ICP-MS). In this analysis, we found zinc was present in Ruc_red_ preparations in close to equimolar amounts; when Ruc_red_ was treated with NEM prior to ICP-MS, little Ruc-bound zinc was detected (Table S1). Taken together, our metal-binding assays demonstrated that cysteine thiols in Ruc coordinate a single zinc atom.

10.1128/mBio.01545-20.5 TABLE S1 Quantification of metals in Ruc by ICP-MS. For each element, the number of spectral counts per second (CPS) was measured, and the concentration of the element was determined using a standard curve (see Materials and Methods). The concentration of sulfur was used to calculate the precise protein concentration in each sample; this was used to determine the molar ratio of Zn to Ruc. Phosphate-buffered saline (PBS) without protein was used to measure background levels of elements in the assay buffer. Download Table S1, XLSX file, 0.01 MB. Copyright © 2020 Becker et al. 2020 Becker et al. This content is distributed under the terms of the Creative Commons Attribution 4.0 International license.

Aside from the N-terminal zinc ribbon fold domain, the entire C-terminal half of Ruc was predicted to lack secondary structure (Fig. 2B). To evaluate the degree of disorder in Ruc, we measured the secondary structures found in Ruc_red_ and Ruc_ox_ using circular dichroism (CD) spectroscopy (reviewed in reference 37). In accordance with structural predictions, both Ruc_red_ and Ruc_ox_ yielded a CD spectrum characteristic of disordered proteins (Fig. 3C) (38).

### Oxidized Ruc prevents unfolded protein aggregation *in vitro*.

**FIG 4:**
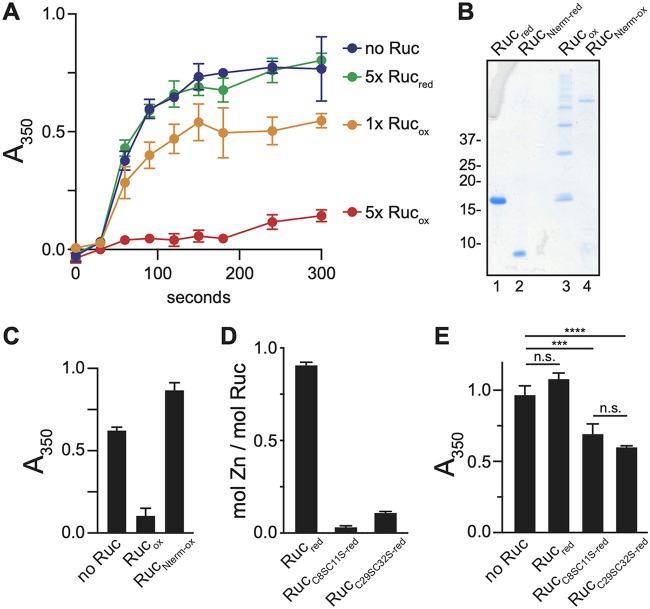
Oxidized Ruc inhibits protein aggregation. (A) Aggregation of luciferase upon heat denaturation. Luciferase was incubated at 45°C either alone or in the presence of a 5-fold molar excess (5×) of Ruc_red_, 5× Ruc_ox_, or an equimolar concentration (1×) of Ruc_ox_. Aggregation was assessed by absorbance at 350 nm (A_350_). The difference in aggregation between no Ruc and 1× Ruc_ox_ or 5× Ruc_ox_ conditions was statistically significant (paired *t* test; *P < *0.01), while no significant difference was obtained with Ruc_red_. (B) Ruc and Ruc_Nterm_ (comprising residues 1 to 49), in either a reduced or oxidized state, were separated on a Coomassie-stained SDS-PAGE gel. (C) Aggregation of heat-denatured luciferase as in panel A, except only the 300-s time point is shown. Native Ruc_ox_ or Ruc_Nterm-ox_ was incubated with luciferase in 5-fold molar excess at 45°C as indicated. (D) Quantification of zinc in native Ruc and Ruc cysteine-to-serine variants, as described for Fig. 3B. NEM was included in all reactions. (E) Luciferase aggregation assay as in panel C to assess the activity of reduced Ruc cysteine-to-serine variants. Statistical significance was determined using one-way ANOVA. ****, *P < *0.0001; ***, *P < *0.001; n.s., not statistically significant (*P > *0.05). All reactions were performed in triplicate.

Chaperones are able to inhibit protein aggregation due to their propensity to bind to unfolded proteins. A common method for detecting chaperone activity uses purified firefly luciferase, which denatures and forms irreversible aggregates when heated above 42°C; the presence of a chaperone during denaturation of luciferase prevents its aggregation (16, 39). We therefore used this method to test chaperone activity by Ruc. When we incubated luciferase at 45°C, we observed the formation of precipitates that could be detected by an increase in light absorbance at 350 nm. In the presence of a 5-fold molar excess of Ruc_red_, we observed a similar level of luciferase aggregation, demonstrating that Ruc_red_ has little to no chaperone activity. In contrast, the presence of Ruc_ox_ during heat inactivation, either in excess or at an equimolar concentration, significantly inhibited luciferase aggregation (Fig. 4A). When we measured luciferase aggregation using a different method of detection, light scattering (40), we also observed the inhibition of aggregation by Ruc_ox_ but not by Ruc_red_ (Fig. S1A). Furthermore, chaperone activity by Ruc was observed when Ruc was pretreated with the oxidizing agents hypochlorite or nitric oxide (Fig. S1B), further supporting a model whereby oxidized Ruc counteracts protein aggregation. Importantly, Ruc was also activated by NEM (Fig. S1C), which prevents disulfide bond formation, demonstrating that the generation of covalently linked Ruc multimers during oxidation (Fig. 3A) is not formally required for Ruc chaperone activity and may be an *in vitro* artifact.

10.1128/mBio.01545-20.1 FIG S1 Ruc_ox_ inhibits protein aggregation and can be activated by multiple oxidants. (A) Measurement of denatured luciferase aggregation using light scattering spectrophotometry. Luciferase was incubated alone in the presence of a 40-fold (40×) or 10-fold (10×) molar excess of Ruc_ox_, or a 40-fold molar excess of Ruc_red_. (B and C) Measurement of denatured luciferase aggregation using absorbance at 350 nm (A_350_). Luciferase was incubated at 45°C for 5 minutes either alone (−) or in the presence of Ruc pretreated with 2 mM DTT, 2 mM H_2_O_2_ with 0.5 mM CuCl_2_, 2 mM diethylamine NONOate (DEANO, a nitric oxide donor), 2 mM sodium hypochlorite (NaOCl), or 2 mM NEM. Data in panel A are representative of two independent experiments; the experiments in panels B and C were performed using three replicates per condition. Statistical significance was determined using one-way ANOVA. ****, *P < *0.0001; *, *P < *0.05; n.s., not statistically significant (*P > *0.05). Download FIG S1, PDF file, 0.2 MB. Copyright © 2020 Becker et al. 2020 Becker et al. This content is distributed under the terms of the Creative Commons Attribution 4.0 International license.

The high degree of disorder present in Ruc, along with our previous observation that all Ruc homologs are predicted to harbor domains that lack secondary structure, supports a hypothesis whereby the unstructured C-terminal domain somehow contributes to the function of this protein. In the activation process of Hsp33, oxidation induces conformational changes that expose a disordered region with a high affinity for client proteins (41, 42). We therefore asked if the intrinsically disordered C-terminal domain of Ruc was required for its chaperone activity. We produced a truncated Ruc variant, Ruc_Nterm_ (amino acids 1 through 49), which harbors only the zinc-binding motif (Fig. 4B, lane 2). In contrast to the variety of multimers observed for Ruc_ox_, oxidized Ruc_Nterm_ (Ruc_Nterm-ox_) formed a single high-molecular-weight species (Fig. 4B, compare lanes 3 and 4). When we tested the chaperone activity of oxidized Ruc_Nterm_ (Ruc_Nterm-ox_), we found that it was unable to prevent luciferase aggregation (Fig. 4C). Thus, the disordered C terminus of Ruc is required for its chaperone activity, either by binding to client protein, by influencing the conformation or oligomerization state of Ruc, or through a combination of factors.

We next asked if replacing the Ruc cysteines, which we predicted would disrupt zinc coordination, would allow for constitutive Ruc activity in the absence of oxidation, a phenomenon that is observed for Hsp33 (34). We made cysteine-to-serine (C-S) substitutions in Ruc, generating Ruc_C8S,C11S_ and Ruc_C29S,C32S_ (see Table 1 for primers and plasmids). Neither Ruc_C8S,C11S-red_ nor Ruc_C29S,C32S-red_ were bound to zinc upon their purification (Fig. 4D). When we tested the ability of each reduced Ruc variant to inhibit luciferase aggregation, we found that both exhibited significant chaperone activity compared to wild-type Ruc_red_ (Fig. 4E). Collectively, these results support a model by which Ruc is in a zinc-bound, inactive state under reducing conditions and that oxidation and zinc release promotes a conformation of Ruc that allows for its interaction with an unfolded protein.

### Oxidized Ruc promotes protein refolding by M. tuberculosis Hsp70/Hsp40 chaperones.

**FIG 5:**
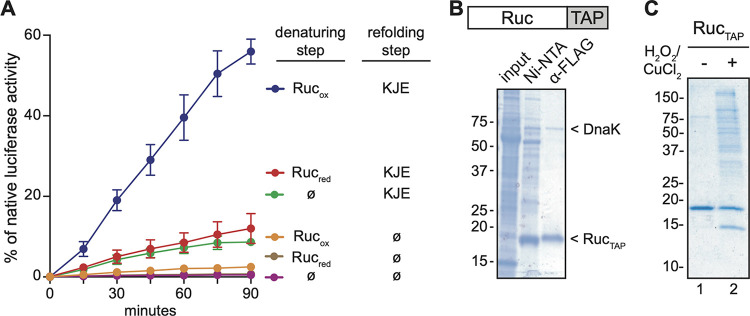
Ruc promotes protein folding by the M. tuberculosis Hsp70 system and associates with many M. tuberculosis proteins. (A) Luciferase was denatured at 45°C in the presence of either Ruc_red,_ Ruc_ox_, or a buffer control (ø). Reactions were then cooled to 25°C and incubated either with DnaK, DnaJ2, and GrpE (KJE). Refolding of denatured luciferase was determined by measuring luciferase activity at the indicated time points following addition of KJE or buffer. As a control, native luciferase activity was measured at each time point by incubating nondenatured luciferase in the same buffer for the same duration (see Materials and Methods for a detailed protocol). Data shown are the result of three independent experiments comparing each condition. (B) Ruc containing a C-terminal Ruc_TAP_ (illustrated above) was purified from M. tuberculosis strain MHD1541. A two-step purification was performed on soluble M. tuberculosis lysates (input) using Ni-NTA resin followed by FLAG antibody gel (α-FLAG). The identity of DnaK was determined using mass spectrometry (see Materials and Methods) and immunoblotting (see Fig. S2A). (C) RucTAP was purified from M. tuberculosis as in panel B, except that input lysates were subjected to oxidation (H_2_O_2_ and CuCl_2_ treatment) or no treatment prior to purification. The final α-FLAG-purified material is shown. For panels B and C, samples were separated on SDS-PAGE gels under reducing conditions.

Non-ATPase bacterial chaperones such as Hsp33 and sHsps prevent irreversible protein aggregation *in vivo*; critically, this function relies on the ability of ATP-dependent chaperones to eventually refold substrates that are bound by these non-ATPase chaperones (5, 6, 43). To further understand the potential role of Ruc in M. tuberculosis protein quality control, we tested if Ruc could promote protein refolding by M. tuberculosis DnaKJE. To test this hypothesis, we heated luciferase either alone or in the presence of Ruc_red_ or Ruc_ox_; after bringing the reaction to room temperature and adding purified M. tuberculosis DnaK, DnaJ2, and GrpE under reducing conditions, we monitored refolding by measuring luciferase activity over time. Only minimal refolding of luciferase by DnaKJE was observed when luciferase was denatured in the absence of a chaperone, a result that was expected based on previous studies (5, 6, 12, 43). In contrast, significant DnaKJE-mediated refolding of luciferase was achieved when luciferase was denatured in the presence of Ruc_ox_ (Fig. 5A). Consistent with its inability to prevent luciferase aggregation (Fig. 4A), Ruc_red_ had no significant effect on luciferase refolding (Fig. 5A). These results demonstrate that unfolded proteins that are bound by Ruc under oxidizing conditions can be refolded by the DnaKJE system.

10.1128/mBio.01545-20.2 FIG S2 Ruc_TAP_ copurifies with DnaK, and subjecting M. tuberculosis to oxidation does not affect Ruc_TAP_ purification. (A) Ruc_TAP_ or PrcB_TAP_ (a control, TAP-tagged protein) purifications from M. tuberculosis were separated on an SDS-PAGE gel and either stained with Coomassie brilliant blue (left) or analyzed by immunoblotting with a monoclonal antibody against M. tuberculosis DnaK (right). (B) Ruc_TAP_ was purified in two steps (Ni-NTA resin followed by α-FLAG affinity gel) from M. tuberculosis cultures (strain MHD1541) that were either untreated or treated with a sublethal concentration of oxidants (0.625 mM H_2_O_2_ with 16 nmol CuCl_2_) for 30 minutes at 45°C. Download FIG S2, PDF file, 0.9 MB. Copyright © 2020 Becker et al. 2020 Becker et al. This content is distributed under the terms of the Creative Commons Attribution 4.0 International license.

After establishing the chaperone activity of Ruc *in vitro*, we sought to better understand the specific role of Ruc in M. tuberculosis physiology by identifying potential protein interaction partners *in vivo*. To capture such interactions, we produced Ruc with a hexahistidine-FLAG tandem affinity purification tag (Ruc_TAP_) in M. tuberculosis. When we performed a two-step purification of Ruc_TAP_ from M. tuberculosis lysates under native conditions, we observed that a second protein copurified with Ruc_TAP_ (Fig. 5B); mass spectrometry identified this protein as DnaK (see Materials and Methods). We validated the identity of DnaK in Ruc_TAP_ purifications by immunoblotting with a monoclonal antibody to DnaK (Fig. S2A). Importantly, this result was specific to Ruc_TAP_, as DnaK did not copurify with another small TAP-tagged protein, PrcB_TAP_ (Fig. S2A). This apparent interaction between Ruc and DnaK in M. tuberculosis lysates supports the hypothesis that Ruc is directly involved in maintaining protein folding by the DnaKJE system *in vivo*.

Notably, Ruc_TAP_ interacted with DnaK under native purification conditions in which no oxidants were added. Given that Ruc chaperone function is activated under oxidizing conditions, we next sought to capture the interaction of Ruc with endogenous client proteins by purifying Ruc_TAP_ from bacteria subjected to oxidative stress, a condition that could promote the association of Ruc_TAP_ with unfolded substrates. However, when we purified Ruc_TAP_ from M. tuberculosis cultures that were exposed to a sublethal concentration of H_2_O_2_ and CuCl_2_ at 45°C, we did not observe any additional proteins copurifying with Ruc_TAP_ (Fig. S2B). We hypothesized that intact M. tuberculosis rapidly reverses oxidation such that interactions between Ruc_ox_ and unfolded client proteins are too transient to capture. We instead treated lysates of Ruc_TAP_-producing M. tuberculosis with H_2_O_2_ and CuCl_2_, and found that oxidation resulted in the association of many M. tuberculosis proteins with Ruc_TAP_ (Fig. 5C, lane 2). To determine the identity of these putative clients of Ruc, we performed proteomic mass spectrometry to identify the proteins that copurified with Ruc_TAP_ under oxidizing, but not native, conditions (Table S2). Proteins that copurified with activated Ruc_TAP_ were associated with a diverse range of functions, including nitrogen and carbon metabolism, electron transport, oxidative stress, and transcriptional regulation; thus, Ruc chaperone activity likely provides a general protective effect on the M. tuberculosis proteome during oxidation. While these results suggest that Ruc can interact with a wide variety of proteins, it remains to be determined if the same interactions take place within bacteria under any condition.

10.1128/mBio.01545-20.6 TABLE S2 M. tuberculosis proteins that copurified with Ruc_TAP_. Proteins are listed by UniProt accession and entry numbers. To compare proteins copurifying with either reduced or oxidized Ruc_TAP_, SAINT scores (see Materials and Methods) were used to calculate the FDR; proteins whose SAINT score yielded an FDR of 5% or lower were considered statistically significant and are highlighted in the table. Download Table S2, XLSX file, 0.1 MB. Copyright © 2020 Becker et al. 2020 Becker et al. This content is distributed under the terms of the Creative Commons Attribution 4.0 International license.

### Ruc is not essential for M. tuberculosis resistance to several oxidative stresses *in vitro*.

During an infection, M. tuberculosis primarily resides within macrophages and neutrophils (44). These immune cells are capable of mounting an antimicrobial response that includes the generation of reactive oxygen species (ROS), reactive nitrogen intermediates (RNI), and hypochlorite. These molecules, all of which can react with cysteine thiols, can confer lethal stress upon infecting pathogens (reviewed in reference 45). Animals defective in the ability to produce ROS, RNI, and hypochlorite are more susceptible to bacterial and fungal pathogens (46–49), and human deficiency in ROS production is associated with susceptibility to mycobacterial infections (50).

Our data thus far led us to hypothesize that Ruc contributes to M. tuberculosis physiology by protecting bacteria from protein aggregation during oxidative stress. Therefore, we sought to test if a *ruc* mutant was more susceptible to a variety of oxidative stress conditions *in vitro*. We challenged M. tuberculosis strains incubated at 45°C with either peroxide, hypochlorite, plumbagin (which generates superoxide radicals in cells) (51), or acidified nitrite (which produces NO) (52) and qualitatively assessed the approximate lethal dose of each compound. The WT and *ruc* mutant strains were equally susceptible to killing under all stress conditions tested (Fig. S3A). We also measured the sensitivity of M. tuberculosis to peroxide stress in a quantitative assay and again observed that the *ruc* mutant was as sensitive to oxidation as the WT strain (Fig. S3B). Thus, in addition to our observation that the *ruc* mutant does not have a significant virulence defect in C57BL/6J mice, we were unable to conclusively determine if Ruc protects M. tuberculosis from the various oxidative stress conditions that might be encountered inside or outside a host. Given that M. tuberculosis has multiple mechanisms for maintaining bacterial redox balance, including thioredoxins, mycothiol, and catalase (reviewed in reference 53), it is possible that the contribution of Ruc to M. tuberculosis fitness only becomes apparent under conditions in which these antioxidant systems are not fully effective. Along these lines, the requirement for Hsp33 for E. coli resistance to oxidative stress was initially observed only after the thioredoxin system was genetically disrupted (17).

10.1128/mBio.01545-20.3 FIG S3 WT and *ruc* strains are equally sensitive to oxidants. (A) A series of two-fold serial dilutions of each oxidant was added to M. tuberculosis at the final concentrations shown; cultures were incubated along with oxidants at 45°C for 4 h. Afterward, cultures were spot plated onto solid agar media, and plates were incubated at 37°C for 2 weeks to assess surviving bacteria. (B) Sensitivity of M. tuberculosis strains to oxidation was quantitatively assessed by incubating bacterial cultures at 45°C with 20 mM H_2_O_2_ and 500 μM CuCl_2_ for the indicated times. Following incubation, cultures were diluted, spread onto solid agar media, and incubated at 37°C for 2 weeks to measure the number of colony forming units (CFU). For panels A and B, MHD1385, MHD1393, and MHD1394 are represented. Download FIG S3, PDF file, 1.1 MB. Copyright © 2020 Becker et al. 2020 Becker et al. This content is distributed under the terms of the Creative Commons Attribution 4.0 International license.

## DISCUSSION

In this study, we identified Ruc as the founding member of a new family of bacterial redox-regulated chaperones. Prior to this work, Hsp33, RidA, and CnoX were the only other proteins described in bacteria whose chaperone activity is dependent on oxidation. Remarkably, however, Ruc shares no sequence similarity to these proteins aside from the presence of four zinc-coordinating cysteines, a feature of Hsp33. Reduced, inactive Hsp33 compactly folds into two globular domains; a combination of high temperature and oxidation causes partial unfolding of the protein, exposing a disordered region with a high affinity for substrates (41, 42). The Hsp33 zinc-binding motif, while essential for inducing conformational changes upon oxidation, does not directly participate in substrate binding (41). In contrast to Hsp33, Ruc likely has a single, small globular region and is predicted to be intrinsically disordered across more than half the length of the protein. We therefore expect that the mechanism of its activation is distinct from that of Hsp33. Our observation that Ruc_red_ has no chaperone activity suggests that the disordered domain of Ruc_red_ is kept in a partially occluded state, perhaps by interacting with other regions of the protein, and would therefore be unavailable for binding a client protein until oxidation takes place. Structural studies of Ruc, both alone and in complex with a substrate, will be necessary to determine the precise mechanism of its activation.

**FIG 6:**
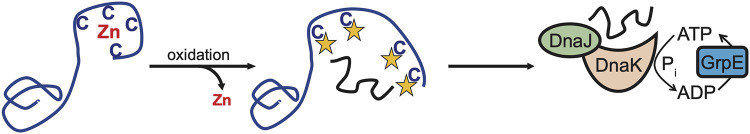
Model of Ruc chaperone activity in M. tuberculosis. From left to right, under the steady-state reducing conditions of the cytoplasm, Ruc coordinates zinc and is inactive, upon oxidation of Ruc cysteines, zinc is displaced and Ruc binds to unfolded proteins, and a Ruc client protein becomes bound to the Hsp70 system for refolding into its native conformation.

It is well established that Hsp33, CnoX, and sHsps deliver unfolded proteins to ATPase chaperones for refolding (5, 6, 22, 43). Here, we describe a similar function of Ruc in promoting protein folding by M. tuberculosis DnaKJE (Fig. 6). Importantly, despite strong evidence that proteins can be directly transferred from non-ATPase to ATPase chaperones, a direct interaction between a holdase and an Hsp70 chaperone has never been observed (15). Thus, our ability to capture the interaction of Ruc and DnaK, even in the absence of environmental stress, may present a new opportunity to understand mechanisms by which holdases transfer their substrates to DnaK.

While oxidized Ruc supports protein folding by DnaKJE, a chaperone system that is essential for M. tuberculosis viability (54), we found that Ruc is dispensable for M. tuberculosis survival under the *in vitro* and *in vivo* conditions tested in this study. However, *ruc* is conserved across pathogenic mycobacteria, including Mycobacterium leprae, a species whose genome has undergone extensive gene loss and is thought to contain only “core” mycobacterial genes (55). Thus, we propose that Ruc is required for M. tuberculosis survival under specific stress conditions that were not fully recapitulated in our experiments. Notably, a previous screen to identify genes required for M. tuberculosis virulence in mice during mixed infections found that bacteria with transposon insertions in *ruc* exhibited approximately 2-fold less growth *in vivo* than growth *in vitro* (56), possibly mirroring the subtle phenotype we observed in our mouse infections. In another study, mice of a different genetic background (BALB/c) that were infected with an M. tuberculosis strain containing a deletion and disruption mutation of Rv0990c (encoding Hsp22.5), the gene directly downstream of *ruc*, had a lower bacterial burden during the later stages of infection and increased time to death than mice infected with a WT strain; however, the phenotypes in this study could not be fully complemented (57). Conceivably, the Rv0990c mutation could have affected Ruc production, which might explain the incomplete complementation of the Rv0990c mutation to fully restore virulence. Furthermore, the phenotype of the Rv0990c mutant was observed at a later time point than those analyzed in this work.

The widespread conservation of Ruc homologs suggests that this protein protects bacteria against one or more common environmental stresses, as is the case for Hsp33. In a comparison of the phyletic distribution of Hsp33 and Ruc (Fig. S4), both proteins are present in certain bacterial lineages but strongly anticorrelated in others. For example, Hsp33 is widespread in *Firmicutes*, *Cyanobacteria*, *Alphaproteobacteria*, and *Gammaproteobacteria* (>80% of species), whereas Ruc is only rarely found in these organisms. In contrast, in lineages such as *Planctomycetes*, *Chloroflexi*, and *Actinobacteria* (including *Mycobacteria*), Hsp33 homologs are rare or absent, while Ruc homologs tend to be dominantly encoded by these genomes (Fig. S4). We therefore speculate that for these species, Ruc fulfills a similar role to that of Hsp33, preventing the irreversible aggregation of unfolded proteins during oxidative stress, a condition in which ATP depletion or direct thiol modification of DnaK may render DnaKJE-mediated refolding impossible (6, 20, 58). If this scenario were indeed supported by further studies, then Ruc and Hsp33, which appear to be structurally unrelated, may represent products of convergent evolution.

10.1128/mBio.01545-20.4 FIG S4 Phyletic distribution of Ruc and Hsp33. Each phylum is plotted based on the percentage of species that contain Hsp33 (*x* axis) and Ruc (*y* axis). Download FIG S4, PDF file, 0.5 MB. Copyright © 2020 Becker et al. 2020 Becker et al. This content is distributed under the terms of the Creative Commons Attribution 4.0 International license.

## MATERIALS AND METHODS

### Strains, plasmids, primers, and culture conditions.

See Table 1 for strains, plasmids, and primers used in this work. Reagents used for making all buffers and bacterial media were purchased from Thermo Fisher Scientific unless otherwise indicated. M. tuberculosis was grown in 7H9c (BD Difco Middlebrook 7H9 broth with 0.2% glycerol and supplemented with 0.5% bovine serum albumin [BSA], 0.2% dextrose, 0.085% sodium chloride, and 0.05% Tween 80). For the experiment in Fig. 1C, bacteria were grown in Proskauer-Beck minimal medium supplemented with asparagine and a similar result was observed in 7H9c. For solid media, M. tuberculosis was grown on 7H11 agar (BD Difco Middlebrook 7H11) containing 0.5% glycerol and supplemented with 10% final volume of BBL Middlebrook OADC enrichment. For selection of M. tuberculosis, the following antibiotics were used as needed: kanamycin 50 μg/ml and hygromycin 50 μg/ml. E. coli was cultured in BD Difco Luria-Bertani (LB) broth or on LB agar. Media were supplemented with the following antibiotics as needed: kanamycin, 100 μg/ml; hygromycin, 150 μg/ml; and ampicillin, 200 μg/ml.

Primers used for PCR amplification or sequencing were purchased from Life Technologies and are listed in Table 1. DNA was PCR amplified using either Phusion (New England Biolabs [NEB]), *Pfu* (Agilent), or *Taq* (Qiagen) according to the manufacturers’ instructions. PCR products were purified using the QIAquick gel extraction kit (Qiagen). Plasmids encoding His_6_-SUMO-Ruc, His_6_-SUMO-Ruc_Nterm_, His_6_-SUMO-Ruc_C8S,C11S_, and His_6_-SUMO-Ruc_C29S,C32S_ were made using splicing by overlap extension (SOE) PCR (59). Restriction enzymes and T4 DNA ligase were purchased from NEB. The following plasmids were made by PCR-amplifying genes from M. tuberculosis DNA using the indicated primers and cloning amplification products into their respective vectors: pET24b(+)-Rv0991c-his_6_ (NdeI-Rv0991c-F, HindIII-Rv0991c-R); pMV306kan-Rv0991c (HindIII-Rv0991c-F, XbaI-Rv0991c-R); pOLYG-Rv0991c-TAP (HindIII-Rv0991c-F, XbaI-Rv0991c-TAP-R); pAJD107-Rv0991c (NdeI-Rv0991c-F, BglII-Rv0991c-R); and pAJD107-Rv0991c-C8S,C11S (NdeI-Rv0991c-C8S,C11S-F, BglII-Rv0991c-R). pET24b(+)-Rv0991c and pET24b(+)-Rv0991c-C8S,C11S were made by subcloning the Rv0991c gene from pAJD107-Rv0991c and pAJD107-Rv0991c-C8S,C11S into pET24b(+), respectively. pET24b(+)-HisSUMO-*ruc* was made by first PCR amplifying HisSUMO from pEcTL02 using primers T7-F and SUMO-Ruc-soeR, second, amplifying *ruc* from M. tuberculosis DNA using SUMO-Ruc-soeF and Ruc-KpnI-R, and, finally, performing SOE PCR using these two amplification products along with primers T7-F and Ruc-Kpn-R. The SOE PCR product was then cloned into pET24b(+). pET24b(+)-HisSUMO-*ruc-*Nterm was made similarly except that the primer RucNterm-KpnI-R substituted for Ruc-KpnI. pET24b(+)-HisSUMO-*ruc-*C29S,C32S was made by SOE PCR using primers T7-F, T7-term, Rv0991c-C29SC32S-F, and Rv0991c-C29SC32S-R, with pET24b(+)-HisSUMO-*ruc* as the PCR template. pET24b(+)-HisSUMO-*ruc-*C8SC11S was made similarly to pET-24b(+)-HisSUMO-*ruc* except that the amplification product from primers SUMO-Ruc-soeF and Ruc-KpnI-R was made using pET24b(+)-Rv0991c-C8S,C11S as a template.

Calcium chloride-competent E. coli DH5α was transformed with ligations. All plasmids were purified from E. coli using the QIAprep spin miniprep kit. All plasmids made by PCR cloning were sequenced by Genewiz, Inc. to ensure the veracity of the cloned sequence. Plasmids were transformed into M. tuberculosis by electroporation as previously described (60). Single-colony transformants were isolated on 7H11 agar with antibiotic selection.

### Protein purification, antibody production, and immunoblotting.

Ruc-His_6_ was produced in E. coli strain ER2566; His_6_-SUMO-Ruc, His_6_-SUMO-RucNterm, His_6_-SUMO-Ruc_C8S,C11S_, His_6_-SUMO-Ruc_C29S,C32S_, His_6_-SUMO-DnaK, His_6_-SUMO-DnaJ2, His_6_-SUMO-GrpE, and His_6_-Ulp1 were produced in E. coli strain BL21. Proteins were purified from E. coli by affinity chromatography using Ni-nitrilotriacetic acid (NTA) agarose (Qiagen) according to the manufacturer’s instructions (Ruc-His_6_ was purified under urea denaturing conditions). Production of His_6_-SUMO-DnaK, His_6_-SUMO-DnaJ2, and His_6_-SUMO-GrpE in E. coli was performed as previously described (12). To make rabbit polyclonal immune serum, approximately 200 μg Ruc-His_6_ was used to immunize rabbits (Covance, Denver, PA).

M. tuberculosis Ruc was prepared from E. coli using two methods that yielded approximately equal purity; both methods also resulted in identical Ruc chaperone activity upon oxidation of the protein. In the first method, untagged Ruc was purified from strain ER2566. A 500-ml culture was grown at 37°C with shaking to an optical density at 600 nm (OD_600_) of 0.5 and then cooled to room temperature. We added 1 mM isopropyl β-d-thiogalactopyranoside (IPTG), and the culture was grown further at 30°C with shaking for 5 h. Bacteria were collected by centrifugation, resuspended in 25 ml of 50 mM Tris, pH 8.0, and lysed by sonication. After removing insoluble material by centrifugation, ammonium sulfate was added to the lysate to 70% wt/vol, and the suspension was stirred for 30 min at 4°C to precipitate proteins. The precipitate was collected by centrifugation, resuspended in 3 ml of 50 mM Tris, pH 8.0, and dialyzed against 4 liters of the same buffer at 4°C overnight to remove residual ammonium sulfate and dissolve proteins. Ruc has a predicted isoelectric point of approximately 9 and was therefore one of the few positively charged proteins in the bacterial protein extract. Taking advantage of this fact, Ruc was purified to homogeneity by passing the protein extract over a Q-Sepharose anion exchange column (GE); Ruc immediately exited the column in flowthrough fractions. To ensure that the protein was fully reduced after purification, we treated Ruc with 5 mM dithiothreitol (DTT) for 30 min at 30°C. An Amicon centrifugal filter unit (Millipore) was used to thoroughly buffer exchange the protein into 50 mM HEPES, pH 7.5. The complete removal of DTT was confirmed by measuring the presence of DTT in the filter flowthrough using Ellman’s reagent (Thermo Scientific) according to the manufacturer’s instructions. The final protein preparation (Ruc_red_) was stored in the same buffer at −20°C.

The second method of preparing Ruc from E. coli used a cleavable affinity tag and was also used to obtain Ruc_Nterm_, Ruc_C8S,C11S_, and Ruc_C29S,C32S_. His_6_-SUMO-Ruc, His_6_-SUMO-Ruc_Nterm_, His_6_-SUMO-Ruc_C8S,C11S_, and His_6_-SUMO-Ruc_C29S,C32S_ were each purified from strain BL21(DE3) in the following manner. A 500-ml culture was grown at 37°C with shaking to an OD_600_ of 0.3 to 0.4, then transferred to a 25°C shaking incubator, and grown to an OD_600_ of 0.5. We added 1 mM IPTG, and the culture was further grown for 5 h. Purified protein was prepared from bacteria using Ni-NTA resin and then exchanged into a SUMO cleavage buffer of 50 mM Tris, 150 mM NaCl, 2 mM DTT, pH 8.0. A 1:100 volume of purified His_6_-Ulp1 (SUMO protease) was added, and the reaction mixture was incubated for 30 min at 30°C. His_6_-Ulp1 and His_6_-SUMO were then removed by incubating the mixture with Ni-NTA resin and saving the supernatant fraction; clearance of tagged protein was performed twice to yield pure, native Ruc and truncation or substitution variants. Proteins were buffer exchanged into Tris-buffered saline (TBS) buffer (50 mM Tris, 150 mM NaCl, pH 8.0) using centrifugal filters, and the absence of residual DTT was confirmed using Ellman’s reagent. The final protein preparations (Ruc_red_, Ruc_Nterm-red_, Ruc_C8S,C11S-red_, and Ruc_C29S,C32S-red_) were stored in TBS at −20°C.

M. tuberculosis DnaK, DnaJ2, and GrpE were prepared by removing the affinity tags from His_6_-SUMO-DnaK, His_6_-SUMO-DnaJ2, and His_6_-SUMO-GrpE in the same manner as described for His_6_-SUMO-Ruc except that the native proteins were buffer exchanged into 50 mM Tris, 150 mM KCl, 20 mM MgCl_2_, and 2 mM DTT, pH 7.5, before storage at −20°C.

Separation of proteins in *in vitro* assays and in M. tuberculosis lysates was performed using 15% sodium dodecyl sulfate-polyacrylamide (SDS-PAGE) gels, with the following exceptions. For Fig. 1B and C, AnykD Mini-Protean TGX precast protein gels (Bio-Rad) were used. Bio-Safe Coomassie stain (Bio-Rad) was used to stain gels. For preparing samples for SDS-PAGE gels in Fig. 3A and Fig. 4B, purified proteins were mixed with 4× nonreducing SDS buffer (250 mM Tris, pH 6.8, 2% SDS, 40% glycerol, and 1% bromophenol blue) to a 1× final concentration, and samples were boiled for 5 min. For Fig. 3A, lane 3, DTT was added to the sample to a 2-mM final concentration prior to boiling. For immunoblots, proteins were transferred from protein gels to nitrocellulose membranes (GE Amersham) and analyzed by immunoblotting as indicated. Due to Ruc’s high isoelectric point, Ruc was transferred to membranes using 100 mM *N*-cyclohexyl-3-aminopropanesulfonic acid (CAPS) buffer in 10% methanol. In Fig. 1B and C, Ruc and PrcB immunoblots were from the same membrane. For detecting M. tuberculosis DnaK, we used a monoclonal antibody from BEI Resources (catalog no. NR-13609) at a concentration of 1:1,000 in 3% BSA in 25 mM Tris-Cl/125 mM NaCl/0.05% Tween 20 buffer (TBST). Polyclonal antibodies against PrcB (25) and Ruc were used similarly. Secondary antibodies horseradish peroxidase (HRP)-conjugated goat anti-rabbit IgG F(ab')_2_ and HRP-conjugated anti-mouse IgG (H+L) were purchased from Thermo Fisher Scientific. All antibodies were made in TBST with 3% BSA. Immunoblots were developed using SuperSignal West Pico Plus chemiluminescent substrate (Thermo Fisher Scientific) and imaged using a Bio-Rad ChemiDoc system.

### Preparation of M. tuberculosis extracts for immunoblotting.

M. tuberculosis cultures were grown to an OD_580_ of 0.3. Equal amounts of bacteria were harvested by centrifugation, resuspended in TBS, and transferred to a tube containing 250 μl of 0.1 mm zirconia beads (BioSpec Products). Bacteria were lysed using a mechanical bead beater (BioSpec Products). Whole lysates were mixed with 4× reducing SDS sample buffer (250 mM Tris, pH 6.8, 2% SDS, 20% 2-mercaptoethanol, 40% glycerol, and 1% bromophenol blue) to a 1× final concentration, and samples were boiled for 5 min. For preparing lysates from M. tuberculosis grown in 7H9, which contains BSA, an additional wash step with phosphate-buffered saline with Tween 20 (PBS-T) was done prior to resuspension of bacteria in lysis buffer.

### Mouse infections.

Six- to eight-week-old female C57BL6/J mice (Jackson Laboratories) were each infected with ∼200 to 400 M. tuberculosis bacilli by the aerosol infection route. The bacterial burden in organs was determined as previously described (61). Briefly, at the time points indicated in the text, lung pairs and spleens were harvested from three to five mice for each experiment and were each homogenized and plated on 7H11 agar to determine CFU per organ. All procedures were performed with the approval of the New York University Institutional Animal Care and Use Committee.

### Preparation of Ruc_ox_ and Ruc_Nterm-ox_.

We diluted 8.8 M H_2_O_2_ stock solution, stored at −20°C, to 200 mM in deionized water just prior to the oxidation reaction. We incubated 100 μM Ruc_red_ or Ruc_Nterm-red_ at 37°C for 3 min, after which 50 μM copper chloride was added, followed by 2 mM H_2_O_2_. The reaction mixture was incubated for 10 min; H_2_O_2_ and copper chloride were removed using a Zeba Spin 7K MWCO desalting centrifuge column (Fisher Scientific) preequilibrated with the original Ruc_red_ storage buffer according to the manufacturer’s instructions. Treatment of Ruc with 2 mM diethylamine NONOate (DEANO, a nitric oxide donor) (Sigma-Aldrich) or NaOCl (Fig. S1B) was performed identically except that Ruc was treated for 20 min (NaOCl) or 2 h (DEANO) as described for Hsp33 (62).

### Spectrophotometric detection of zinc in Ruc preparations.

Quantification of the zinc coordinated by Ruc cysteines was performed using 4-(2-pyridylazo)resorcinol (PAR) using a previously established method (34, 63) except that zinc-cysteine complexes were disrupted using *N*-ethylmaleimide (NEM) (Pierce) according to the manufacturer’s instructions. We mixed 25 μM Ruc with 100 μM PAR either in the absence or presence of 2 mM NEM. Reaction mixtures were incubated at room temperature for 1 h. Zn(PAR)_2_ complexes were detected by A_500_ using a NanoDrop spectrophotometer. To obtain a precise concentration of zinc in protein preparations, serial dilutions of zinc sulfate prepared in matched buffers (with or without NEM) were used to prepare standard curves. Three technical replicates were performed for each condition.

### Metal analysis of Ruc preparations using ICP-MS.

Elemental quantification of purified Ruc with and without NEM and a buffer control was performed using an Agilent 7700 inductively coupled plasma mass spectrometer (Agilent, Santa Clara, CA) attached to a Teledyne Cetac Technologies ASX-560 autosampler (Teledyne Cetac Technologies, Omaha, NE). The following settings were fixed for the analysis: cell entrance, −40 V; cell exit, −60 V; plate bias, −60 V; OctP bias, −18 V; and collision cell helium flow, 4.5 ml/min. Optimal voltages for extract 2, omega bias, omega lens, OctP RF, and deflect were determined empirically before each sample set was analyzed. Element calibration curves were generated using Aristar ICP standard mix (VWR, Radnor, PA). Samples were introduced by peristaltic pump with 0.5-mm internal diameter tubing through a MicroMist borosilicate glass nebulizer (Agilent). Samples were initially taken at 0.5 revolutions per second (rps) for 30 s followed by 30 s at 0.1 rps to stabilize the signal. Samples were analyzed in spectrum mode at 0.1 rps, collecting three points across each peak and performing three replicates of 100 sweeps for each element analyzed. Sampling probe and tubing were rinsed for 20 s at 0.5 rps with 2% nitric acid between every sample. Data were acquired and analyzed using the Agilent Mass Hunter workstation software version A.01.02.

### CD spectrophotometry.

CD measurements were performed using a Jasco J-1500 CD spectrophotometer as per the manufacturer's instructions. To prepare samples, Ruc was buffer exchanged into 20 mM KH_2_PO_4_, pH 7.5, diluted to 20 μM, and transferred to a quartz cuvette. The spectrophotometer parameters were set as follows: CD scale, 200 millidegrees (mdeg)/1.0 delta optical density; integration time, 1 s; and bandwidth, 1 nm. The voltage was monitored simultaneously and remained below 700 V.

### Luciferase aggregation and refolding assays.

The chaperone activity of Ruc was measured by testing its ability to limit the aggregation of heat-denatured luciferase, a previously established method (16, 39). For experiments in Fig. 4A, C, and E, aggregation was determined by measuring absorbance at 350 nm (A_350_) (64, 65). Firefly luciferase (Promega) was diluted to 2 μM in TBS and mixed in a microcentrifuge tube with concentrations of Ruc indicated in the text and figures, or TBS alone, to a final volume of 20 μl. The tube was then incubated in a 45°C heat block. Immediately before incubation and at the indicated times, a 2-μl volume was removed, and A_350_ was measured using a NanoDrop spectrophotometer. Aggregation assays were performed using three technical replicates per condition. For the experiment in Fig. S1A, luciferase aggregation was measured using light scattering as previously described (16).

Refolding of heat-denatured luciferase by M. tuberculosis DnaKJE was performed using a protocol adapted from reference 12. M. tuberculosis encodes two DnaJ homologs, DnaJ1 and DnaJ2, which both promote DnaK-mediated protein folding *in vitro* (12); DnaJ2 was used in this study because we found that DnaJ1 exhibited poor solubility when purified from E. coli. For the denaturation step, luciferase was diluted to 0.1 μM in TBS, either alone or mixed with 4 μM Ruc_red_ or Ruc_ox_, in glass vials. Denaturation was performed by placing vials in a 45°C water bath for 20 min. For the refolding step, we used glass-coated 96-well plates in which 5 μl of denaturation reaction was mixed with 15 μl of refolding reaction buffer (50 mM Tris pH 7.5, 150 mM KCl, 20 mM MgCl_2_, 2 mM DTT, 1 mg/ml BSA [Sigma-Aldrich], and 2 mM Mg^2+^-ATP) or refolding reaction buffer containing 4 μM DnaK, 2 μM DnaJ2, and 2 μM GrpE. Plates were incubated at 25°C. Luciferase activity was measured immediately upon initiating the refolding step (0 min) and at all other time points indicated in Fig. 5A by transferring 2 μl of each reaction into a white opaque 96-well plate and adding 100 μl of luciferase assay mix (100 mM KH_2_PO_4_, pH 7.5, 25 mM glycyl glycine, 0.2 mM EDTA, 2 mM Mg^2+^-ATP, 0.5 mg/ml BSA, and 70 μM luciferin). Luminescence was measured using a plate reader (PerkinElmer EnVision). For calculating the percentage of native luciferase activity for all reactions at each time point, a control reaction was included in which 0.1 μM luciferase was diluted into TBS in a glass vial but kept at 4°C, rather than heated, during the denaturation step. This control reaction was mixed with refolding reaction buffer as described above, and luminescence was measured at each time point in Fig. 5A to determine 100% native luciferase activity.

### Tandem affinity purification of M. tuberculosis proteins.

Purifications of TAP-tagged proteins from M. tuberculosis were performed under low-salt conditions as described (66). The following changes were made to the protocol: 100 μl of packed Ni-NTA beads and 100 μl of M2 anti-FLAG affinity gel were used; 100 μl of 100 μM 3 × FLAG peptide was used for the final elution. For capturing Ruc_TAP_ interactions with other M. tuberculosis proteins, M. tuberculosis lysates were incubated at 45°C for 10 min either in the absence (Fig. 5C, lane 1) or presence (Fig. 5C, lane 2) of 2 mM H_2_O_2_ and 50 μM CuCl_2_; purifications were subsequently performed as described above. Samples were boiled in 4× reducing SDS sample buffer prior to running SDS-PAGE gels.

### Protein mass spectrometry.

To identify the ∼70-kDa protein pulled down by Ruc_TAP_ (Fig. 5B), the band was excised from SDS-PAGE gel and processed as previously described (67). To determine the identity of proteins enriched in Ruc_TAP_ purifications under oxidizing conditions (Fig. 5C; Table S2), Ruc_TAP_ purifications under native or oxidizing conditions were performed in three biological replicates. Affinity-purified samples were reduced, alkylated, digested with trypsin, and desalted as previously described (25, 67). The peptide eluates in all cases were concentrated in the SpeedVac and stored at −80°C. Aliquots of each sample were loaded onto a trap column (Acclaim PepMap 100 precolumn, 75 μm by 2 cm, C18, 3 μm, 100 Å; Thermo Scientific) connected to an analytical column (Easy-Spray column, 50 m by 75 μm ID, PepMap RSLC C18, 2 μm, 100 Å, Thermo Scientific) using the autosampler of an Easy-nLC 1000 (Thermo Scientific) with solvent A consisting of 2% acetonitrile in 0.5% acetic acid and solvent B consisting of 80% acetonitrile in 0.5% acetic acid. The peptide mixture was gradient eluted into the Orbitrap Q Exactive HF-X mass spectrometer (Thermo Scientific) using the following gradient: 5% to 35% solvent B in 120 min and 35% to 45% solvent B in 10 min followed by 45% to 100% solvent B in 20 min. The full scan was acquired with a resolution of 45,000 (at *m/z* 200), a target value of 3E^6^, and a maximum ion time of 45 ms. Following each full MS scan, 20 data-dependent tandem mass spectrometry (MS/MS) spectra were acquired. The MS/MS spectra were collected with a resolution of 15,000 (at *m/z* 200), an automatic gain control (AGC) target of 1E^5^, maximum ion time of 120 ms, one microscan, 2 *m/z* isolation window, fixed first mass of 150 *m/z*, dynamic exclusion of 30 s, and normalized collision energy (NCE) of 27. All acquired MS^2^ spectra were searched against a UniProt M. tuberculosis H37Rv database, including common contaminant proteins using Sequest HT within Proteome Discoverer 1.4 (Thermo Fisher Scientific). The search parameters were as follows: precursor mass tolerance, ±10 ppm; fragment mass tolerance, ±0.02 Da; digestion parameters, trypsin allowing two missed cleavages; fixed modification of carbamidomethyl on cysteine; variable modification of oxidation on methionine; and variable modification of deamidation on glutamine and asparagine and a 1% peptide and protein false discovery rate (FDR) searched against a decoy database. The results were filtered to only include proteins identified by at least two unique peptides. Fold change analysis was performed for the Ruc_TAP_ purifications using the ratios of peptide spectral matches (PSMs) in oxidized samples to the PSMs in the native affinity-purified samples using the SAINT algorithm (68). SAINT scores were used to calculate the FDR; proteins whose SAINT score yielded an FDR of 5% or lower were considered statistically significant and are highlighted in Table S2.

### Measurement of M. tuberculosis susceptibility to oxidants.

M. tuberculosis was grown in 7H9c to an OD_580_ of 0.5 at 37°C, centrifuged and resuspended in fresh 7H9c, and spun at 500 × *g* to remove clumps of bacteria. Supernatants were then diluted to an OD_580_ of 0.025, transferred to 96-well plates, and incubated at 45°C for 4 h to induce Ruc production. Afterward, M. tuberculosis strains were subjected to oxidizing reagents and inoculated onto 7H11 agar as described in the legend for Fig. S3.

### Computational analyses.

Iterative sequence profile searches were performed to recover Ruc sequence homologs using the PSI-BLAST program (69). Searches were either run against the nonredundant (nr) protein database of the National Center for Biotechnology Information (NCBI) or a custom database of 7,423 complete prokaryotic genomes extracted from the NCBI RefSeq database (70). The latter was used for phyletic profile analyses (Table 2). Contextual information from prokaryotic gene neighborhoods was retrieved using a Perl script that extracts the upstream and downstream genes of a query gene from a GenBank genome file. This was followed by clustering of proteins using the BLASTCLUST program (ftp://ftp.ncbi.nih.gov/blast/documents/blastclust.html) to identify conserved gene neighborhoods. Analysis and visualization of phyletic patterns were performed using the R language.
