# Supplementary figures and images for: Mycobacterium tuberculosis Rv0991c Is a Redox-Regulated Molecular Chaperone
Source: mBio. 2020 Aug 25;11(4):e01545-20. doi: 10.1128/mBio.01545-20 (PMC7448276; doi:10.1128/mBio.01545-20)

**A**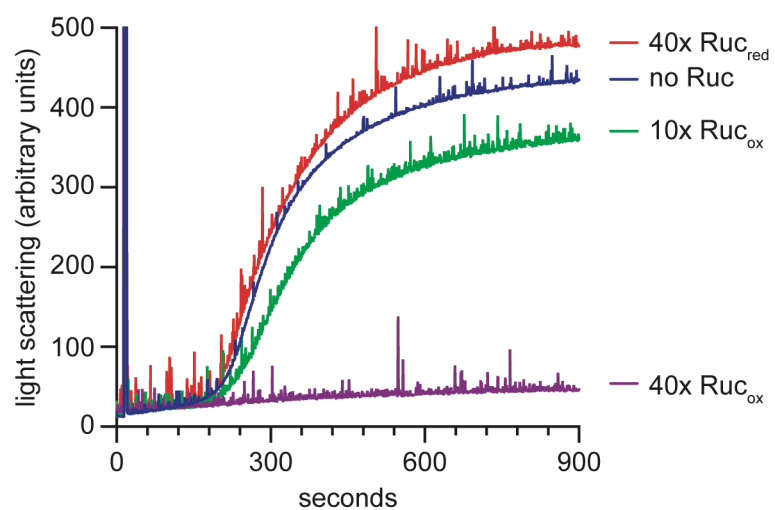**B**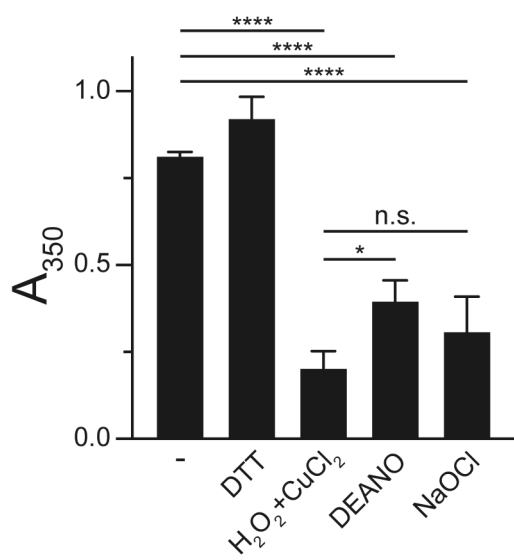**C**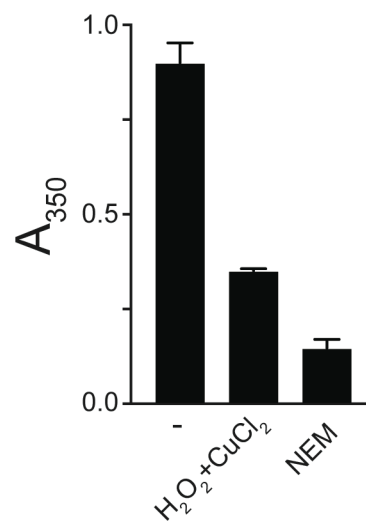

Supplement: FIG S1 [file mBio.01545-20-sf001.pdf]

**A**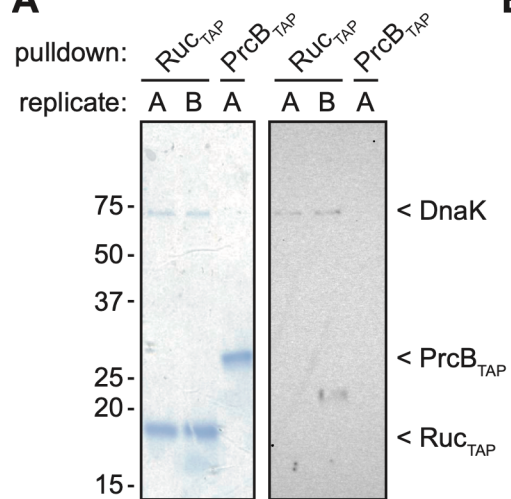**B**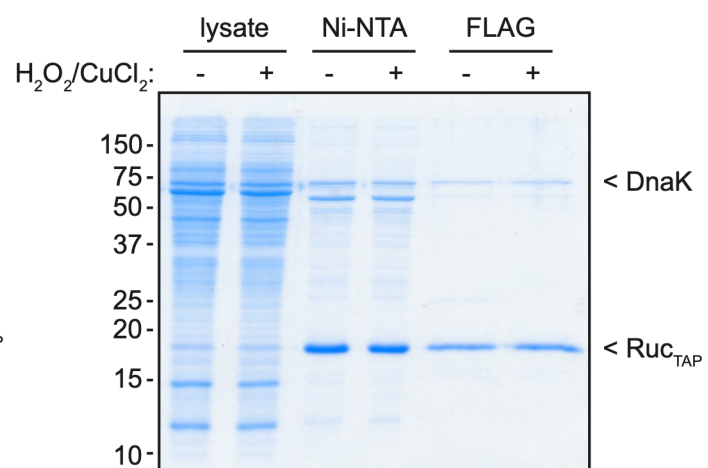

Supplement: FIG S2 [file mBio.01545-20-sf002.pdf]

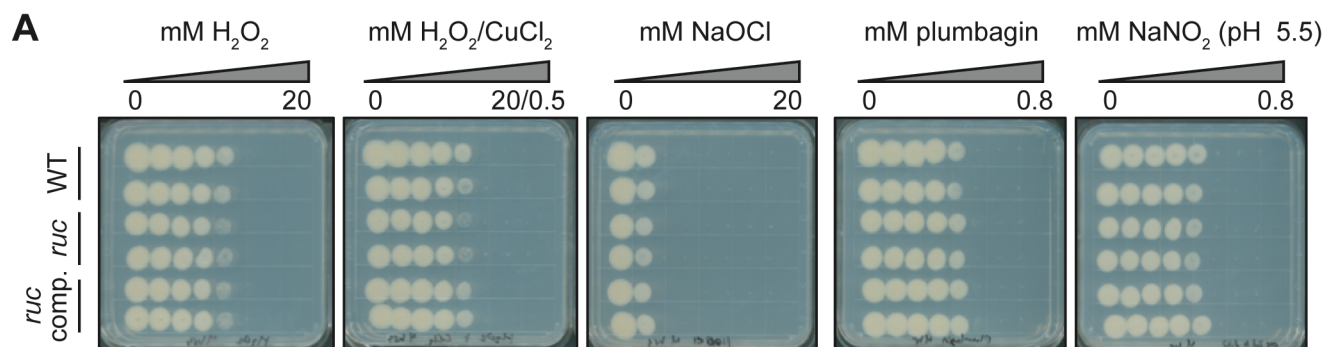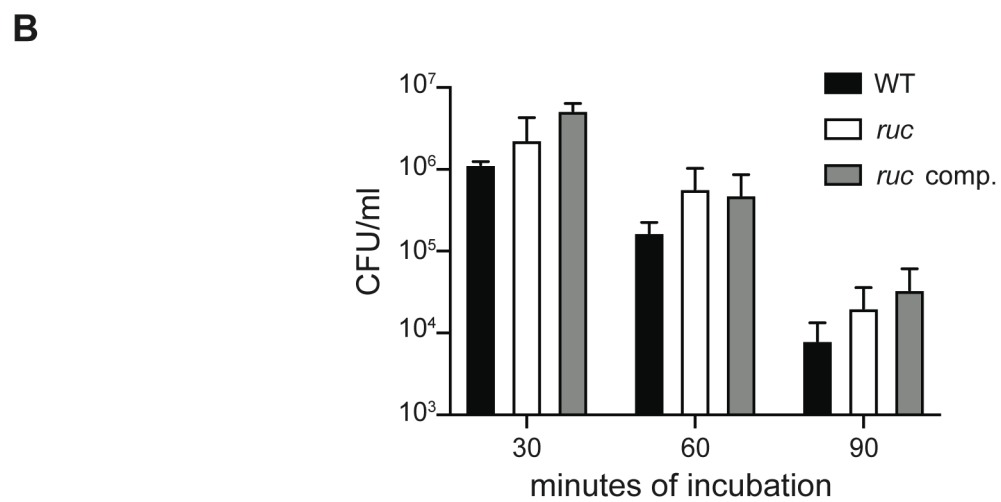

Supplement: FIG S3 [file mBio.01545-20-sf003.pdf]

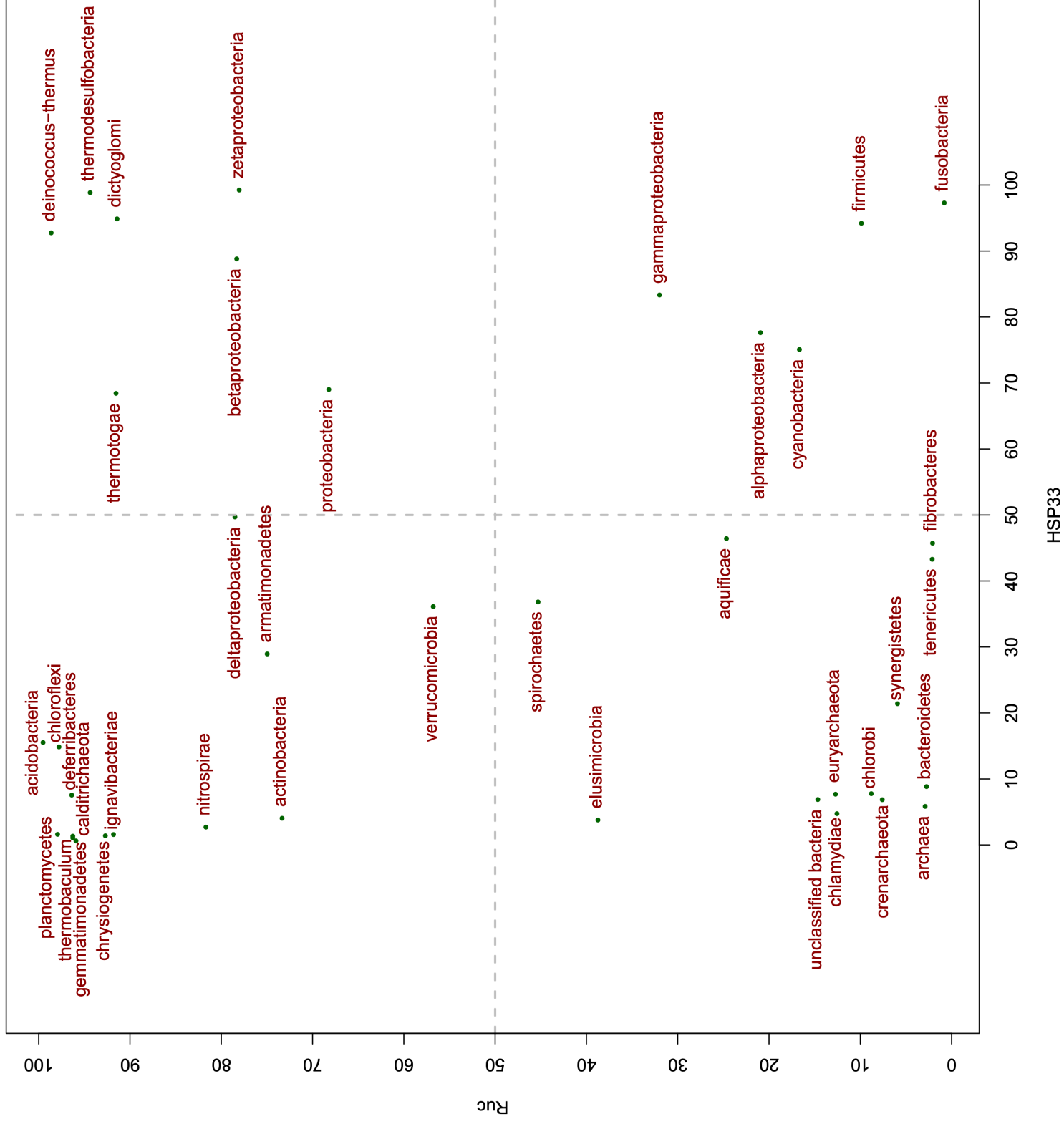

Supplement: FIG S4 [file mBio.01545-20-sf004.pdf]
